# Recurrent *Plasmodium falciparum* Malaria Infections in Kenyan Children Diminish T-Cell Immunity to Epstein Barr Virus Lytic but Not Latent Antigens

**DOI:** 10.1371/journal.pone.0031753

**Published:** 2012-03-12

**Authors:** Cynthia J. Snider, Stephen R. Cole, Kiprotich Chelimo, Peter Odada Sumba, Pia D. M. MacDonald, Chandy C. John, Steven R. Meshnick, Ann M. Moormann

**Affiliations:** 1 Department of Epidemiology, Gillings School of Global Public Health, University of North Carolina, Chapel Hill, North Carolina, United States of America; 2 Center for Global Health Research, Kenya Medical Research Institute, Kisumu, Kenya; 3 Departments of Pediatrics and Medicine, University of Minnesota Medical School, Minneapolis, Minnesota, United States of America; 4 Center for Global Health and Diseases, Case Western Reserve University, Cleveland, Ohio, United States of America; 5 Departments of Pediatrics and Quantitative Health Services, University of Massachusetts Medical School, Worcester, Massachusetts, United States of America; Karolinska Institutet, Sweden

## Abstract

*Plasmodium falciparum* malaria (*Pf*-malaria) and Epstein Barr Virus (EBV) infections coexist in children at risk for endemic Burkitt's lymphoma (eBL); yet studies have only glimpsed the cumulative effect of *Pf*-malaria on EBV-specific immunity. Using pooled EBV lytic and latent CD8+ T-cell epitope-peptides, IFN-γ ELISPOT responses were surveyed three times among children (10 months to 15 years) in Kenya from 2002–2004. Prevalence ratios (PR) and 95% confidence intervals (CI) were estimated in association with *Pf*-malaria exposure, defined at the district-level (Kisumu: holoendemic; Nandi: hypoendemic) and the individual-level. We observed a 46% decrease in positive EBV lytic antigen IFN-γ responses among 5–9 year olds residing in Kisumu compared to Nandi (PR: 0.54; 95% CI: 0.30–0.99). Individual-level analysis in Kisumu revealed further impairment of EBV lytic antigen responses among 5–9 year olds consistently infected with *Pf*-malaria compared to those never infected. There were no observed district- or individual-level differences between *Pf*-malaria exposure and EBV latent antigen IFN-γ response. The gradual decrease of EBV lytic antigen but not latent antigen IFN-γ responses after primary infection suggests a specific loss in immunological control over the lytic cycle in children residing in malaria holoendemic areas, further refining our understanding of eBL etiology.

## Introduction

*Plasmodium falciparum* (*Pf*) malaria and Epstein Barr Virus (EBV) have been identified as co-factors in the pathogenesis of endemic Burkitt's lymphoma (eBL) [1] which is estimated to account for 70% of cancers among children in equatorial Africa [2], [3]. In areas with intense perennial malaria transmission (holoendemic), the highest incidence of eBL is in children aged 4–8 years [4], [5], [6], [7], [8], [9], in contrast to areas with low malaria transmission (hypoendemic) where eBL is rarely reported [7], [10], [11].

It has been hypothesized that *Pf*-malaria infections promote eBL in two mutually-compatible ways. In developing countries, most children experience primary EBV infection by 3 years of age, followed by life-long infection in memory B-lymphocytes [12], [13]. *P. falciparum* induces polyclonal B-cell expansion and lytic EBV reactivation [14], thus increasing the number of latently-infected B-cells. In otherwise healthy individuals, interferon-gamma (IFN-γ) secreting cytotoxic CD8+ T-cells mediate immunosurveillance of EBV [5], [12], [15], [16], [17], [18]. Repeated *Pf*-malaria infections could hence lead to exhaustion or hypo-responsiveness of EBV latent or lytic antigen CD8+ T-cells, thus increasing the chance for this EBV-associated malignancy to arise.

Limited evidence supports an impaired EBV-specific T-cell response in association with *Pf*-malaria. Using an in vitro regression assay as a measure of cytotoxicity, children with acute *Pf*-malaria demonstrated a transient loss of control over B-cell outgrowth [19], [20], [21]. Furthermore, case-control studies comparing acutely *Pf*-malaria infected individuals with healthy adults came to the same conclusion [22], [23]. However, the cumulative effect of repeated often asymptomatic *Pf*-malaria infections on EBV persistence has not been thoroughly studied [1], [6], [24], [25]. Two ecological studies provide the minimum understanding we have on the relationship. A study among adults found a loss of EBV-specific T-cell control among those exposed to holoendemic compared to hypoendemic malaria [26]. A second study only found significantly lower EBV latent and lytic antigen IFN-γ responses in children 5–9 years old residing in the holoendemic area compared to other age groups and children from a hypoendemic area [16].

The objective of this study was to examine the influence of cumulative *Pf*-malaria on EBV latent and lytic antigen CD8+ T-cell IFN-γ ELISPOT responses in children over a two-year period.

## Results

### Participant summary

**Figure 1:**
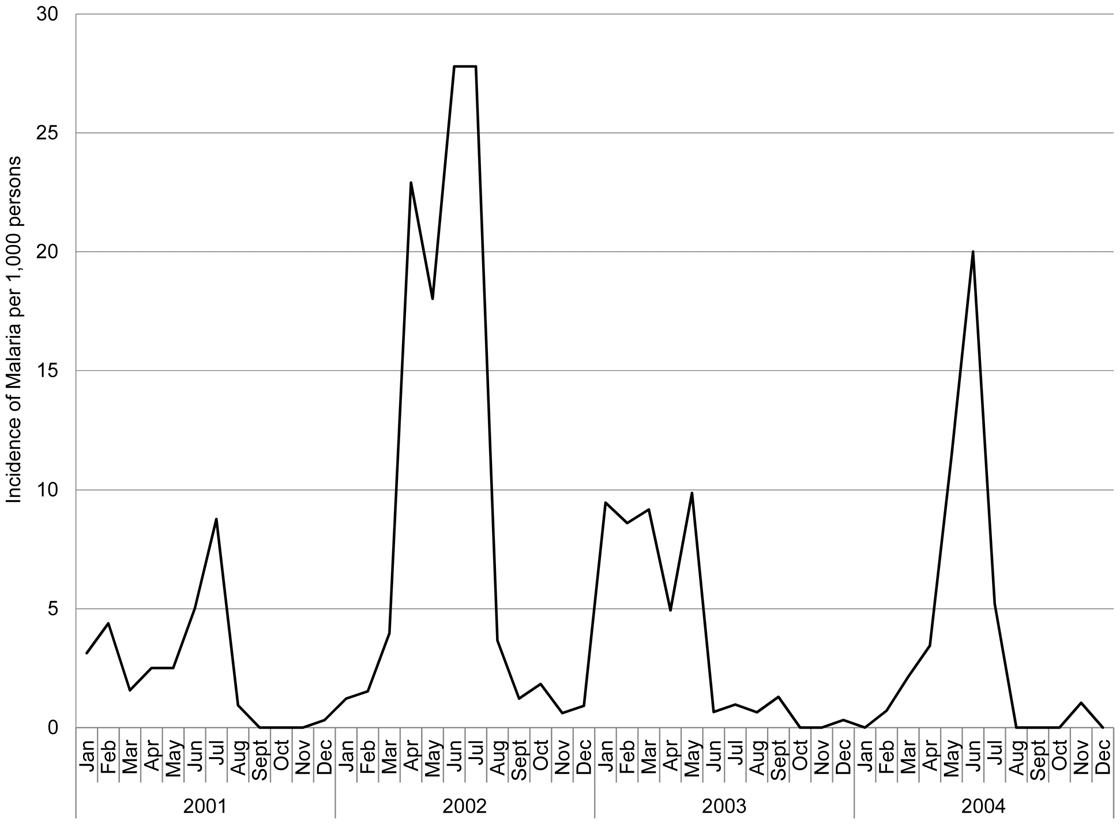
Malaria incidence in the highland area of Kipsamoite, 2001–2004.

Of the 236 children enrolled, 230 (97.5%) were seropositive for EBV [27]. Our weighted analysis included 149 children who participated in all surveys and had interpretable EBV-specific T-cell responses (Table 1). The age and sex distribution between the districts were not significantly different (*P* = .11 and *P* = .30, respectively). Children in Kisumu experienced more *Pf*-malaria infections than children in Nandi (*P*<.001); only 3% of Kisumu children were never infected compared to 78% in Nandi. This was despite a classically defined malaria outbreak in Nandi during the survey periods (Figure 1).

**Table 1:** Summary of participants in the Kisumu/Nandi cohort, Kenya 2002–2004^a^.

	Site				Total
	Kisumu (holoendemic)		Nandi (hypoendemic)		
	*n*	%	*n*	%	
Sex					
Male	39	59.1	38	45.8	77
Female	27	40.9	45	54.2	72
Age (in years)					
0–4	16	24.2	30	36.1	46
5–9	33	50.0	35	42.2	68
≥10	17	25.8	18	21.7	35
Malaria infections					
All surveys	38	57.6	0	0	38
Two surveys	20	30.3	4	4.8	24
One survey	6	9.1	14	16.9	20
Never	2	3.0	65	78.3	67
Total	66		83		149

*n*, number; %, percentage.

a Data in the table are weighted according to the 149 children who participated in all surveys and had interpretable Epstein-Barr virus (EBV) specific CD8+ T-cell IFN-γ response.

#### The magnitude of EBV-specific IFN-γ responses did not differ significantly by malaria endemicity

The proportion of positive IFN-γ responses to PHA (positive control) demonstrates that children from both districts were equally able to elicit an IFN-γ response indicating no global signs of immune dysfunction (Table 2). There were no significant differences in median values of EBV lytic or latent CD8+ T-cell IFN-γ responses between children of similar age groups across districts. Therefore, *Pf*-malaria exposure does not appear to influence the magnitude of EBV-specific IFN-γ responses.

**Table 2:** EBV-specific CD8+ T-cell IFN-γ Response by Residence and Age Group^a^.

	EBV lytic antigens			EBV latent antigens			PHA^b^	
	*n*	%	Median^c^	*n*	%	Median^c^	*n*	%
			(range)			(range)		
Baseline (July–August 2002)								
Kisumu								
0–4 years	7/16	43.8	96 (14–166)	8/16	50.0	43 (20–98)	13/16	81.3
5–9 years	8/33	24.2	67 (18–170)	6/33	18.2	47 (16–448)	31/33	93.9
≥10 years	5/17	29.4	150 (20–350)	5/17	29.4	46 (16–404)	15/17	88.2
Nandi								
0–4 years	12/30	34.3	98 (28–836)	8/30	26.7	70 (18–146)	28/30	93.3
5–9 years	15/35	42.9	50 (22–792)	11/35	31.4	84 (42–668)	33/35	94.3
≥10 years	8/18	22.9	53 (36–304)	8/18	44.4	88 (26–1322)	18/18	100
First follow-up (February–March 2003)								
Kisumu								
0–4 years	4/16	25.0	46 (40–128)	2/16	12.5	55 (32–78)	16/16	100
5–9 years	1/33	3.0	20 (20)	6/33	18.2	15 (14–132)	31/33	93.9
≥10 years	5/17	29.4	30 (18–162)	2/17	11.8	23 (18–28)	16/17	94.1
Nandi								
0–4 years	6/30	20.0	98 (24–744)	4/30	13.3	77 (32–128)	26/30	86.7
5–9 years	8/35	22.9	82 (16–1742)	8/35	22.9	58 (20–248)	34/35	97.1
≥10 years	5/18	27.8	54 (32–382)	3/18	16.7	54 (14–354)	18/18	100
Second follow-up (July–August 2004)								
Kisumu								
0–4 years	5/16	31.3	76 (30–84)	2/16	12.5	106 (64–148)	15/16	93.8
5–9 years	3/33	9.1	60 (56–150)	3/33	9.1	42 (24–74)	33/33	100
≥10 years	3/17	17.7	250 (40–288)	1/17	5.9	16 (16)	17/17	100
Nandi								
0–4 years	8/30	26.7	50 (14–384)	2/30	6.7	69 (58–80)	25/30	83.3
5–9 years	7/35	20.0	76 (14–278)	6/35	17.1	59 (14–214)	31/35	88.6
≥10 years	5/18	27.8	26 (14–130)	5/18	27.8	56 (22–122)	18/18	100

*n*, number; %, percentage; EBV, Epstein-Barr Virus; PHA, Phytohemagglutinin.

a Data in the table are weighted according to the 149 children who participated in all surveys and had interpretable Epstein-Barr Virus (EBV) specific CD8+ T-cell IFN-γ response.

b Phytohemagglutinin (PHA) was used as a positive control.

c Median EBV-specific CD8+ T-cell IFN-γ responses were calculated among children with positive responses and is expressed as spot forming units (SFU) per 1×10^6^ peripheral blood mononuclear cells (PBMC).

### *Pf*-malaria exposure (district-level) and EBV-specific T-cell IFN-γ responses

#### EBV lytic antigen CD8+ T-cell IFN-γ responses

**Figure 2:**
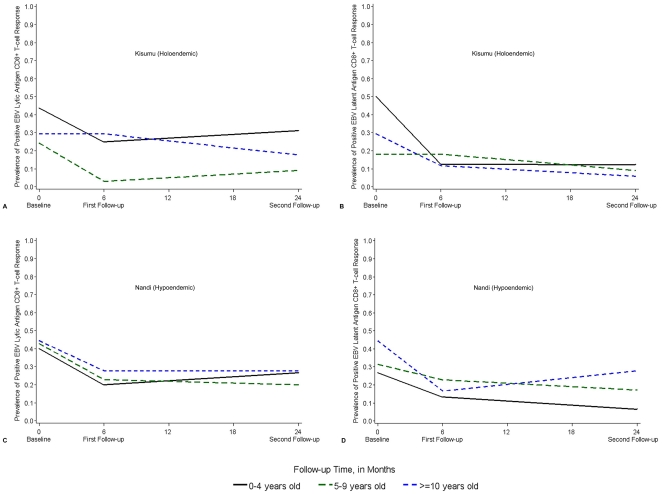
Change in prevalence of EBV-specific CD8+ T-cell IFNγ responses with age. Changes in prevalence of positive EBV lytic (A and C) and latent (B and D) antigen CD8+ T-cell IFNγ response from 2002–2004. Age group at each survey period is based on age at baseline. In Kisumu: 16 (0–4 years), 33 (5–9 years) and 17 (≥10 years). In Nandi: 30 (0–4 years), 35 (5–9 years) and 18 (≥10 years). Solid black line: 0–4 year olds; hash-mark green line: 5–9 year olds; and dotted blue line: ≥10 year old children.

We observed a few intriguing patterns in the prevalence of positive EBV lytic antigen CD8+ T-cell IFN-γ response when children were stratified into age groups by their baseline age (age group cohorts) (Figure 2A,C). In Kisumu, the prevalence of positive responses in the 0–4 year and 5–9 year cohorts decreased from baseline to first follow-up, but remained unchanged in the ≥10 year cohort. By the second follow-up, responses increased among the 0–4 and 5–9 year cohorts while responses decreased in ≥10 year cohort. However, children in the 5–9 year cohort had the lowest prevalence at each survey period. In Nandi, responses declined in all age group cohorts from baseline to first follow-up and remained almost unchanged in the 5–9 year and ≥10 year cohorts by the second follow-up. In the 0–4 year cohort, however, responses increased. The patterns and prevalence of responses among the age group cohorts were similar at all survey periods, varying <10%.

**Figure 3:**
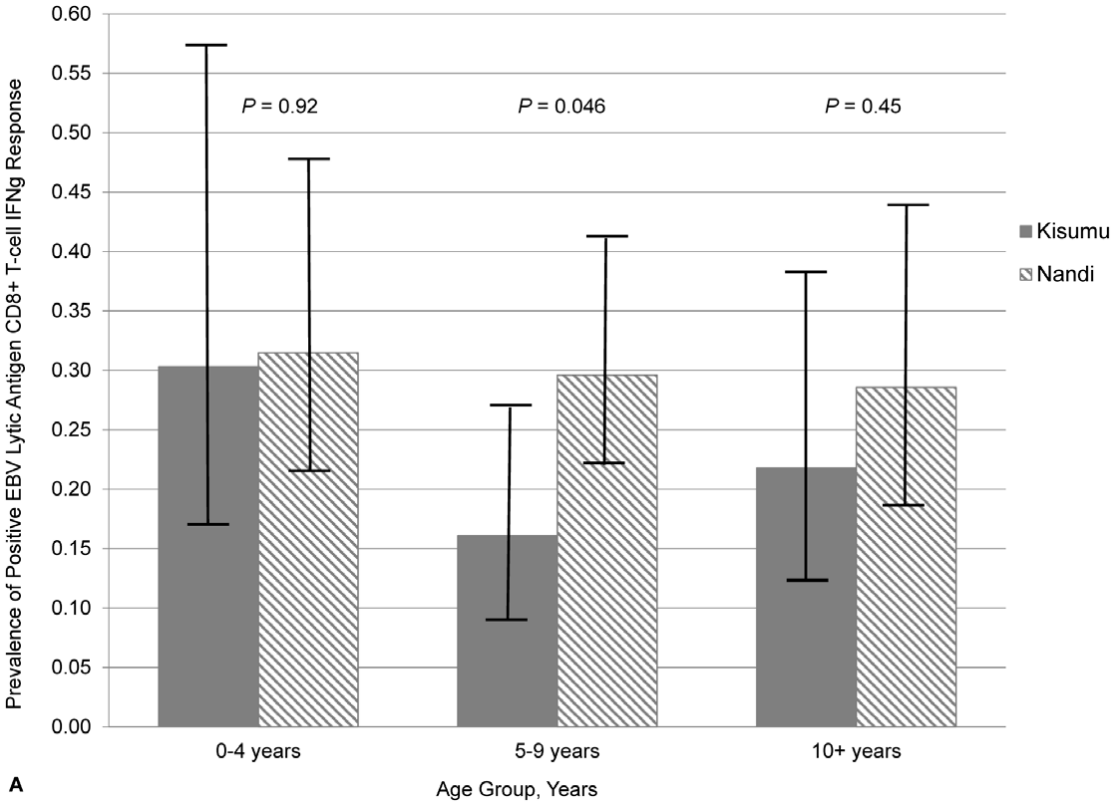
Prevalence of EBV-specific CD8+ T-cell IFNγ response by age group and residence. Prevalence of positive EBV lytic (A) and latent (B) antigen CD8+ T-cell IFNγ response by age group and site of residence, Kenya 2002–2004. Age group was classified as a time-varying factor. For both graphs, the number of observations for children in each age group in Kisumu was: 33 (0–4 years), 87 (5–9 years) and 78 (≥10 years). The number of observations for children in each age group in Nandi was: 54 (0–4 years), 125 (5–9 years) and 70 (≥10 years). *P* values for differences between areas of residence by age group are indicated.

Using the district-level definition of *Pf*-malaria and the weighted model, we estimated the prevalence of positive responses in Kisumu was 0.70 (95% CI: 0.45–1.08) times the prevalence in Nandi although this 30% difference was not significant. In Kisumu, there were no significant differences in positive responses in children 0–4 years (PR: 1.39, 95% CI: 0.60–3.20) and 5–9 years (PR: 0.74, 95% CI: 0.37–1.48) when compared to children ≥10 years (Figure 3A). Likewise in Nandi, the prevalence of positive responses in children 0–4 years (PR: 1.10, 95% CI: 0.60–2.02) and 5–9 years (PR: 1.04, 95% CI: 0.61–1.76) did not differ significantly from children ≥10 years. When similar age groups were compared between districts, we detected a significant difference in children 5–9 years where the prevalence of positive responses in Kisumu was 0.54 (95% CI: 0.30–0.99) that of children in Nandi (Figure 3A). No other differences by age group were found.

#### EBV latent antigen CD8+ T-cell IFN-γ responses

Examining the patterns in the prevalence of positive EBV latent antigen CD8+ T-cell IFN-γ response by age group cohorts, there was variation within and between districts (Figure 2B,D). In Kisumu, the prevalence at baseline was highest among the 0–4 year cohort but then decreased to nearly the same prevalence as the other age group cohorts. In Nandi, there was a decreasing trend from baseline to second follow-up for the 0–4 year and 5–9 year cohorts. However, the ≥10 years cohort had the highest prevalence of response at baseline that decreased by the first follow-up but rebounded by the second follow-up.

From our weighted model, we observed the prevalence of positive responses in Kisumu was 0.80 (95% CI: 0.51–1.25) times the prevalence in Nandi, although not significant. In Kisumu, the prevalence of positive responses in children 0–4 years (PR: 1.93, 95% CI: 0.91–4.13) and 5–9 years (PR: 1.22, 95% CI: 0.61–2.45) was not significantly different from children ≥10 years, although there was a decrease in prevalence with increasing age group (Figure 3B). Similarly in Nandi, responses among children 0–4 years (PR: 0.72, 95% CI: 0.35–1.48) and 5–9 years (PR: 0.84, 95% CI: 0.47–1.49) did not differ significantly from children ≥10 years old, although there was a slight increase in response with increasing age. Despite these interesting trends, there were no significant differences in the prevalence of positive responses when similar age groups were compared between districts.

### *Pf*-malaria infection (individual-level) and EBV-specific T-cell IFN-γ responses

#### EBV lytic antigen CD8+ T-cell IFN-γ responses

Using the individual-level definition of *Pf*-malaria in our weighted model, we found the association between recurrent *Pf*-malaria infections and EBV lytic antigen CD8+ T-cell IFN-γ response varied by age group and survey period. We therefore used two weighted models. In the first model, we stratified results by age group, while adjusting for sex and survey period. Similarly in the second model, we stratified by survey period while adjusting for sex and age group.

We noted three observations from our analysis. First, the PR of recurrent *Pf*-malaria infections and positive IFN-γ responses among Kisumu children were consistently lower than Nandi children for all age groups and survey periods (Table 3). In general, there is a two-fold difference in the PR between Kisumu and Nandi although not significant (*P* = .32). Secondly, the association between recurrent *Pf*-malaria infections and IFN-γ responses varied by age group. In both Kisumu and Nandi, the prevalence of positive responses among children 0–4 years with recurrent *Pf*-malaria infections was higher than that of similarly aged children never infected. In Nandi, the difference was statistically significant. Finally, the PR of recurrent *Pf*-malaria infections and IFN-γ responses to EBV lytic antigens varied by survey period in both districts. At baseline, for both districts, the PR of positive responses among children with recurrent *Pf*-malaria infections was greater compared to children never infected; this result was statistically significant in Nandi, but not Kisumu. However, the PR decreased at subsequent study periods; the prevalence of positive responses among children with recurrent *Pf*-malaria infection diminished over time compared to children never infected. This could reflect functional diminishment of responsive EBV lytic antigen T-cells under continuous pressure from *Pf*-malaria.

**Table 3:** Prevalence Ratios for EBV Lytic Antigen CD8+ T-cell IFN-γ Response.

	Kisumu		Nandi	
	Constant *Pf*-malaria infection versus no infection		Constant *Pf*-malaria infection versus no infection	
	PR	95% CI	PR	95% CI
Unadjusted	0.64	0.23–1.77	1.43	0.73–2.81
Age groups^a^				
0–4 years	1.31	0.28–6.18	3.00	1.72–5.23
5–9 years	0.53	0.15–1.88	1.16	0.39–3.45
≥10 years	0.78	0.16–3.53	0.98	0.33–2.95
Survey periods^b^				
Baseline	1.24	0.49–3.11	1.76	1.07–2.91
Six months	0.29	0.05–1.62	0.73	0.17–3.22
Two years	0.21	0.05–0.92	0.22	0.02–3.23

*Pf*-malaria, *Plasmodium falciparum* malaria; EBV, Epstein-Barr virus; PR, prevalence ratio; CI, confidence interval; Ref, referent group.

a Adjusted for sex and survey period. Unstratified estimates for constant *Pf*-malaria infections compared to never infected in Kisumu (*P* = 0.72) and Nandi (*P* = 0.97) were not significant. Specific details on the number and prevalence of positive responses for each age group are included in Table 2.

b Adjusted for sex and age group. Unstratified estimates for constant *Pf*-malaria infections compared to never infected was not significant in Kisumu (*P* = 0.65) but significant in Nandi (*P* = 0.03). The number of children in Kisumu for each survey period was 66 and the number of children in Nandi was 83.

#### EBV latent antigen CD8+ T-cell IFN-γ responses

Using our weighted model, we did not observe any variation by age group (Table 4) or survey period (data not shown). In Kisumu, for all age groups, the adjusted prevalence of positive EBV latent antigen CD8+ T-cell IFN-γ response was higher among children with recurrent *Pf*-malaria infections compared to those never infected (Table 4). There was a two-fold difference in the PR for children 0–4 years and >10 years with recurrent *Pf*-malaria infections than children 5–9 years. In Nandi, children 0–4 years with recurrent *Pf*-malaria infections had fewer positive responses than children never infected, and a PR that was three-fold lower than older children. However, children in older age groups with recurrent *Pf*-malaria infections had higher positive responses than similarly aged children never infected. Despite estimates for Kisumu and Nandi being imprecise and not statistically significant, the observations suggest that children 5–9 years in Kisumu are unable to mount the type of T-cell response as younger and older children. Meanwhile, in Nandi, the increasing PR with age may reflect how a maturing immune system, not continuously exposed to *Pf*-malaria, is able to induce a T-cell response to latent antigens even when co-infected with *Pf*-malaria.

**Table 4:** Prevalence Ratio for EBV Latent Antigen CD8+ T-cell IFN-γ Response.

	Kisumu		Nandi	
	Constant Pf-malaria infection versus no infection		Constant Pf-malaria infection versus no infection	
	PR	95% CI	PR	95% CI
Unadjusted	1.60	0.37–6.92	1.54	0.71–3.35
Age groups^a^				
0–4 years	2.10	0.22–19.65	0.51	0.08–3.37
5–9 years	1.14	0.26–4.99	1.47	0.58–3.63
≥10 years	2.68	0.38–18.73	1.82	0.83–3.99

*Pf*-malaria, *Plasmodium falciparum* malaria; EBV, Epstein - Barr virus; PR, prevalence ratio; CI, confidence interval.

a Adjusted for sex and survey period. Unstratified estimates for constant *Pf*-malaria infections compared to never infected in Kisumu (*P* = 0.32) and Nandi (*P* = 0.13) were not significant. Specific details on the number and prevalence of positive responses for each age group are included in Table 2.

## Discussion

Our study demonstrates that the prevalence of positive EBV lytic- but not latent-antigen CD8+ T-cell IFN-γ responses decreases in a malaria holoendemic area and not a hypoendemic area. This suggests that children repeatedly infected with *Pf*-malaria eventually lose functional IFN-γ producing CD8+ T-cells in response to EBV lytic antigens. In an effort to control viral replication induced by recurrent *Pf*-malaria infections [14], we hypothesize that EBV lytic antigen CD8+ T-cells have become exhausted and unable to produce IFN-γ or alternatively these cells were culled through apoptosis. As a result of the loss of responsive EBV lytic antigen CD8+ T-cells, more B-lymphocytes could become latently infected by EBV, and thus gradually increasing the risk of eBL. These findings are consistent with previous studies of this cohort, which detected significantly higher median EBV viral load and EBV-specific IgG antibodies to EBV lytic and latent antigens in the holoendemic compared to hypoendemic area [27],[28].

Furthermore, the association between *Pf*-malaria infections and positive EBV lytic antigen CD8+ T-cell IFN-γ responses varied by age group. The EBV lytic antigen deficiency was most pronounced among children 5–9 years old in the malaria holoendemic area and was further potentiated in those recurrently infected with *Pf*-malaria. In our individual-level analysis, these children had the lowest PR of positive responses while this same age group in the hypoendemic area appeared to be affected little. Additionally, the patterns observed in the age group cohorts clearly showed that the 5–9 year cohort in Kisumu had the lowest prevalence of positive responses among all age group cohorts, in both districts, at each survey period. The sustained inability to produce an effective EBV lytic antigen CD8+ T-cell IFN-γ response among 5–9 year olds may be an etiologically relevant event in eBL development since eBL is most often diagnosed in this age group. Finally, the inconsistency of patterns between age group cohorts within a district suggests there is an age-dependent interaction between *Pf*-malaria and EBV-specific T-cell response. Studies of immune mechanisms that induce exhaustion or deletion are needed to understand maintenance of EBV-specific T-cell immunity, especially in children. In support of this premise is the observation that the ELISPOT responses in both groups of children were lower than those described for healthy adults [29] and our Kenyan adult controls (data not shown). To our knowledge there have been no studies of EBV-specific T-cell immunity in healthy children from non-malaria endemic countries. However our studies of malaria-specific immunity also demonstrate an age-associated instability in cytokine recall responses more pronounced in children compared to adults [30], [31].

This study is an important early step to understanding the cumulative effect of *Pf*-malaria infections on EBV-specific T-cell immunity over time. Availability of data over two-years permitted identification of potentially important biological and environmental mechanisms that only became apparent over time. For example, the association between *Pf*-malaria infection and positive EBV lytic antigen CD8+ T-cell IFN-γ responses varied by age group and survey period. The variation noted with age group is expected because there is an age-dependent increase in T-cell immunity as children develop protection against *Pf*-malaria after repeated infections [32]. Children in malaria holoendemic areas acquire immunity to *Pf*-malaria and EBV during the first years of life, and ongoing studies will compare the development of *Pf*-malaria to EBV-specific T-cell memory. An impairment of EBV-specific T-cell control with progressive EBV reactivation has been described in HIV-infected individuals [33], lending support to a sequential series of events in the etiology of eBL.

Using data, collected during a two-year period, also allowed us to use an individual-level definition for *Pf*-malaria infections. Unlike other studies, our definition accounted for the cumulative effect of *Pf*-malaria infection which has been hypothesized to be critical in the pathogenesis of eBL, rather than the transient effect typically observed with acute *Pf*-malaria infection [1]. However, our definition was vulnerable to misclassification because *Pf*-malaria infection was assessed only twice during the two-year follow-up. Therefore, we may not have captured participants' malaria histories accurately. This misclassification was likely to be differential because children in the holoendemic area were exposed to *Pf*-malaria parasites at a higher frequency, averaging two malaria infections per year, than children in the hypoendemic area [34]. Therefore, we may have underestimated or overestimated the PR for *Pf*-malaria infections and EBV-specific T-cell responses in the holoendemic area.

A strength of our study was the use of two definitions for *Pf*-malaria: 1) district-level according to malaria transmission intensity, and 2) individual-level based on measured *Pf*-malaria infection. Although our findings of EBV lytic antigen CD8+ T-cell IFN-γ responses were consistent with both definitions, our findings of EBV latent antigen CD8+ T-cell IFN-γ responses were inconsistent. This may have been due to the limited power or an underestimation of the influence of *Pf*-malaria infections in hypoendemic areas. However, it also highlights the potential pitfall in attributing district-level results to the individual, also known as the ecological fallacy. The inconsistency may have been due to other factors that differed between the districts and unrelated to malaria transmission intensities. Therefore, we conclude that the use of malaria transmission intensity as a surrogate for malaria infection has been informative yet future studies should endeavor to prospectively collect *Pf-*malaria and EBV co-infections information from individuals to more accurately describe this complex relationship.

There were several potential confounders that were not captured in our study, specifically HIV status, nutritional status, schistosomiasis infection, and socioeconomic status. However, we do not believe the absence of these confounders materially affected our findings. When data were collected in western Kenya from 2002–2004, HIV testing in infants was conducted only when medically warranted. All children enrolled in this study were examined by a clinician and had no obvious signs of illness or malnourishment, and no deaths were reported as of 2009. Therefore, even though it was possible that an underlying HIV infection might influence the rate of malaria parasitaemia [35] the likelihood that a significant number of undiagnosed children remained in this study was low. Schistosomiasis infection was unmeasured yet an examination of the *Pf*-malaria and EBV response relationship indicated adjusting for schistosomiasis infection would have biased our analysis. Finally, is socioeconomic status had been measures, participants and their families would likely have been classified as low socioeconomic status because the main occupation was fishing (Kisumu) and farming (Nandi) in both rural study areas with homes constructed of locally available materials.

Our findings on EBV lytic antigen CD8+ T-cell IFN-γ responses were consistent with the studies that have used residence area (malaria transmission intensity) to explore the cumulative effect of *Pf*-malaria infections on EBV-specific T-cell response. We observed fewer positive EBV lytic antigen CD8+ T-cell IFN-γ responses among 5–9 year old than older children [16]. We also identified a reduction in EBV-specific T-cell response among children living in a holoendemic compared a hypoendemic area [26]. The consistency of our findings with previous studies is important given our limited sample size and precision. Meanwhile, our analysis of EBV lytic antigen CD8+ T-cell IFN-γ response at the individual-level supports findings from previous studies that used residence area as a surrogate for *Pf*-malaria infection.

However, we did not detect the same statistically significant district-level difference in positive EBV latent antigen CD8+ T-cell IFN-γ responses among 5–9 year olds as a previous study [16]. This discrepancy may be due to the limited power of our study. Furthermore, the difference between our individual-level analysis and the previous study may also be due to the use of a surrogate definition of *Pf*-malaria.

This study design marks a step toward examining the individual-level association of *Pf*-malaria infections and EBV-specific T-cell IFN-γ responses and identifies a potential difference between children recurrently infected with *Pf*-malaria compared to children never infected. To adequately quantify this effect, a longitudinal study should be considered which could accurately measure *Pf*-malaria infection and changes in *Pf*-malaria and EBV-specific T-cell immunity over time. The temporal aspects of future studies will be vital to elucidating the precise mechanism by which repeated *Pf*-malaria infections affect EBV persistence and immunity.

## Materials and Methods

The Kisumu/Nandi cohort has been previously described [27]. In brief, the cohort consists of 236 children, randomly selected and between 10 months and 15 years at enrollment, from two districts in western Kenya with disparate *Pf*-malaria transmission intensities: Kisumu is characterized as holoendemic and Nandi as hypoendemic. Due to the age-related incidence of eBL, an equal distribution of children by age and sex were enrolled from each area: children 0–4 years have an elevated risk of eBL whereas 5–9 year olds are at highest risk and ≥10 years old have the lowest risk. Data were collected from 2002–2004 using a standardized survey. Three face-to-face interviews were conducted at baseline (July–August 2002), six month follow-up (February–March 2003), and two-year follow-up (July–August 2004). Blood was also collected for malaria and EBV testing.

*Pf*-malaria infection was confirmed on thick and thin blood smears by microscopy. Testing of EBV-specific T-cell response by IFN-γ ELISPOT has been previously described [16]. Lytic (BRLF1, BZLF1, and BMLF1) and latent (Epstein-Barr nuclear antigen [EBNA] 3A, EBNA 3B, and EBNA3C) antigens were selected and pooled for testing. One positive control (mitogen phytohemmagglutinin [PHA]) was used to stimulate wells and a negative control (phosphate buffer saline [PBS]) was used to measure background IFN-γ response in unstimulated wells. Assays were condensed into a three-week period using the same reagents and personnel to minimize inter-assay variability. Cytotoxic T-lymphocyte (CTL) ImmunoSpot scanning and imaging software (version 4; Cellular Technology Ltd, Shaker Heights, OH) was used to count the number of spot-forming units (SFU) per well; results were expressed as SFU per million peripheral blood mononuclear cells (PBMC). Using a two-sided Fisher's exact test (*P*<.05), EBV lytic and latent epitope-peptide CD8+ T-cell IFN-γ responses were categorized as positive or negative. A positive response was recorded if the proportion of SFUs in the stimulated well was significantly different from the proportion of SFU in the unstimulated well. The magnitude of response was calculated by subtracting the SFU in PBS wells (negative control) from the SFU in the stimulated wells. The median value for the negative control wells was 4 SFU per million PBMCs (range 0 to 772 SFU/million PBMC). Median values were calculated among positive responders only.

Analyses were restricted to EBV seropositive children at baseline [27]. We used two definitions of cumulative *Pf*-malaria. First, *Pf*-malaria exposure was defined according to the malaria transmission intensity of the district (district-level definition): Kisumu (holoendemic) or Nandi (hypoendemic). Next, *Pf*-malaria infection was defined as the cumulative average of *P. falciparum* infection (parasitemia) in a participant over the three survey periods (individual-level definition). The value ranged from 0 (never infected) to 1 (always infected); results and discussion focus on children who were always infected (referred to as recurrent) and never infected. With the individual-level definition, we also included the covariates age group, district, sex, and when the survey was conducted (referred to as survey period) in the analysis. We first examined covariates as potential effect measure modifiers using an a priori cutoff of *P* = .20. In the absence of evidence of effect measure modification, we included covariates in the model as potential confounders.

For descriptive analyses, we used the Chi-square statistic to measure associations between categorical exposures and outcomes. We used the two-sided Wilcoxon rank sum (Mann-Whitney *U*)/Kruskal Wallis test for continuous outcomes. For multivariable analyses, we used weighted log-binomial regression with robust variances to estimate the prevalence ratios (PR) and corresponding 95% confidence intervals (CI). We used generalized estimating equations (GEE) with robust variance estimators to account for correlation due to repeated measurements. A weighted model with inverse probability weights (IPWs) was used to address missing observations due to children not participating in all surveys. When using IPW, observations are assumed to be missing at random; missing data are dependent on observed data but independent of unobserved data [36]. To calculate IPWs, the probability of participation was modeled using available predictor variables. The inverse of the predicted probabilities were calculated and assigned to each individual with complete data. Individuals with complete data were weighted to represent individuals with similar characteristics who have missing data.

To calculate the predicted probabilities for our study, we used logistic regression with first order interaction terms using the following equation:
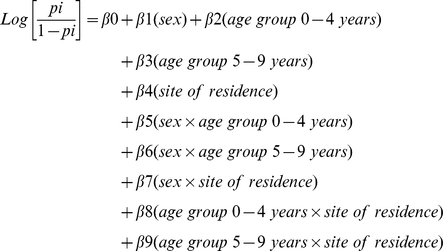
Where p_i_ was the probability of child *i* (*i* = 1,2,3…n) participating in all surveys. Taking the inverse of the predicted probabilities, the mean IPW was 1.44 and ranged from 1.14–1.92. Children who participated in all three surveys were assigned the mean IPW value whereas children with missing observations were assigned an IPW of 0. We also conducted complete case analyses and found no differences in the PR or 95% CI; therefore we report results from the weighted analyses. Data were analyzed in SAS 9.1.3 (Cary, NC).

Written informed consent was obtained from a parent or guardian of the participant. This study was approved by the Institutional Review Boards at the University Hospitals of Cleveland, Case Western Reserve University where Dr. Moormann was affiliated at the time this study was done and also obtained from the Ethical Review Committee for the Kenya Medical Research Institute. It was deemed exempt by the Institutional Review Board at the University of North Carolina at Chapel Hill.
