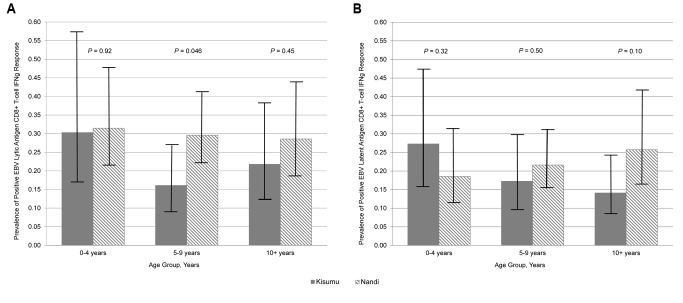# Correction: Recurrent *Plasmodium falciparum* Malaria Infections in Kenyan Children Diminish T-Cell Immunity to Epstein Barr Virus Lytic but Not Latent Antigens

**DOI:** 10.1371/annotation/87d81085-b796-4295-9468-030300d78cd6

**Published:** 2012-06-07

**Authors:** Cynthia J. Snider, Stephen R. Cole, Kiprotich Chelimo, Peter Odada Sumba, Pia D. M. MacDonald, Chandy C. John, Steven R. Meshnick, Ann M. Moormann

The published Figure 3 was incomplete. The complete Figure 3 can be viewed here: 

**Figure:**